# Synergistic interplay of UV radiation and urban particulate matter induces impairment of autophagy and alters cellular fate in senescence‐prone human dermal fibroblasts

**DOI:** 10.1111/acel.14086

**Published:** 2024-01-12

**Authors:** Lena Guerrero‐Navarro, Pidder Jansen‐Dürr, Maria Cavinato

**Affiliations:** ^1^ Institute for Biomedical Aging Research, Universität Innsbruck Innsbruck Austria; ^2^ Center for Molecular Biosciences Innsbruck (CMBI) Innsbruck Austria

**Keywords:** air pollution, apoptosis, autophagy impairment, mitochondrial dysfunction, senescence, skin aging, UV

## Abstract

Skin aging is a complex process influenced by intrinsic factors and environmental stressors, including ultraviolet (UV) radiation and air pollution, among others. In this study, we investigated the effects of UVA and UVB radiation, combined with urban particulate matter (UPM), on human dermal fibroblasts (HDF). We show here that treatment of HDF with a subcytotoxic dose of UVA/UVB results in a series of events leading to mitochondrial dysfunction, increased ROS levels, and DNA damage. These effects are known to trigger either cellular senescence or cell death, depending on the cells' ability to clear damage by activating autophagy. Whereas UPM treatment in isolation did not affect proliferation or survival of HDF, of note, simultaneous UPM treatment of UV‐irradiated cells selectively inhibited autophagic flux, thereby changing cell fate of a fraction of the cell population from senescence to apoptotic cell death. Our findings highlight the synergistic effects of UV radiation and UPM on skin aging, emphasizing the need to consider these factors in assessing the impact of environmental stressors on human health and opening opportunities for developing comprehensive approaches to protect and preserve skin integrity in the face of growing environmental challenges.

## Graphical abstract

Differential Impact of UV Alone, Combined UV, and Urban Particulate Matter on Human Dermal Fibroblasts. Skin aging, influenced by both intrinsic and extrinsic factors, is a complex process involving the interplay of genetics, environmental exposures, and lifestyle choices. UV radiation and air pollution, particularly urban particulate matter, are major external contributors known to accelerate skin aging by triggering cellular damage and inflammatory responses, emphasizing the need to understand their combined effects for effective understanding of their role in the process of skin aging. Here we show that UV exposure leads to mitochondrial fragmentation and DNA damage. Despite the incurred damage, UV‐irradiated cells maintain their ability to engage in autophagy effectively. This active autophagic flux is crucial for mitigating damage, preventing the escalation to widespread apoptotic cell death, and managing senescence levels. Simultaneous exposure to UV radiation and urban particulate matter (UPM) exacerbates mitochondrial fragmentation and DNA damage. Crucially, UV + UPM inhibits autophagic flux, impairing the cell's ability to clear damage. This impairment pushes a greater fraction of the cell population from a senescence state towards apoptosis, suggesting a synergistic effect that may accelerate the skin aging process more significantly than UV alone.

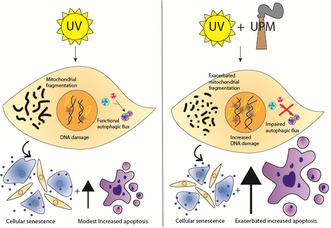

**Abbreviations**
FACS: fluorescence activated cell
FIS1: fission, mitochondrial 1
GAPDH: glyceraldehyde‐3‐phosphate dehydrogenase
HDF: human dermal fibroblasts
IL‐1: interleukin 1
MFN1: mitofusin 1
MMP‐1: matrix metalloproteinase‐1
ROS: reactive oxyigen species
RT‐qPCR: reverse transcription quantitative polyimerase chain reaction
SA‐β‐gal: senescence‐associated beta galactosidase
Ser15: serine 15
UPM: urban particulate matter
UV: ultraviolet

Aging is a complex process involving progressive deterioration of tissues and organs, leading to a decline in organismal functions and the onset of different diseases (López‐Otín et al., 2023). The exposome encompasses the harmful elements to which the skin is exposed, such as pathogens, pollutants, and UV radiation (Krutmann et al., 2017). Exposure of the skin to various exposome factors leads to the accumulation of senescent fibroblasts within the dermal layers, an important factor driving the onset of premature skin aging (Wang & Dreesen, 2018). Repeated exposure to UVA or UVB alone and to a combination of these wavelengths causes cumulative damage and promotes cellular senescence in skin fibroblasts, melanocytes, and keratinocytes (Cavinato & Jansen‐Dürr, 2017; Debacq‐Chainiaux et al., 2012; Wang & Dreesen, 2018). Air pollution, which consists of gases, urban particulate matter (UPM) and, other harmful substances (Kampa & Castanas, 2008), accelerates skin aging through ROS‐induced cellular damage (Dijkhoff et al., 2020; Martic et al., 2022).

A significant proportion of the global population lives in heavily polluted regions, potentially leading to compromised health span as individuals age. However, our understanding of the underlying mechanisms elicited upon the combined exposure of the skin to UV radiation and air pollution and their contribution to skin aging is still limited. Therefore, it is essential to analyze the joint impacts of these stressors given that previous research on extrinsic aging mainly focused on single stressors in isolation, thereby failing to accurately represent the complexity of real‐world conditions.

In this study, we investigated the effects of UV (UVA + UVB) radiation, alone and in combination with urban particulate matter (UPM), on human dermal fibroblasts (HDF). Cells were subjected to daily treatments for four consecutive days using UPM, UVA plus UVB (UV), or a combination of both (UV + UPM). The cells were maintained in culture for a total of 15 days (Figure 1a), while untreated HDF was used as control (Greussing et al., 2013). We observed no significant impact on the proliferation of HDF exposed to UPM alone in comparison to untreated controls. However, upon exposure to UV radiation, the proliferative capacity of fibroblasts was substantially reduced, as was previously shown for UVB. Intriguingly, the simultaneous exposure to UV + UPM resulted in decreased cell numbers throughout the growth curve (Figure 1b), suggesting that UPM synergizes with UV irradiation to either reduce the rate of proliferation or to induce cell death. Under UV and UV + UPM conditions, there was an increase in the G1 phase and a decrease in S phase in comparison to untreated controls and UPM‐treated cells, with no significant difference between UV and UV + UPM, indicating a similar percentage of cells in the cycling fraction (Figure S1A).

**FIGURE 1:**
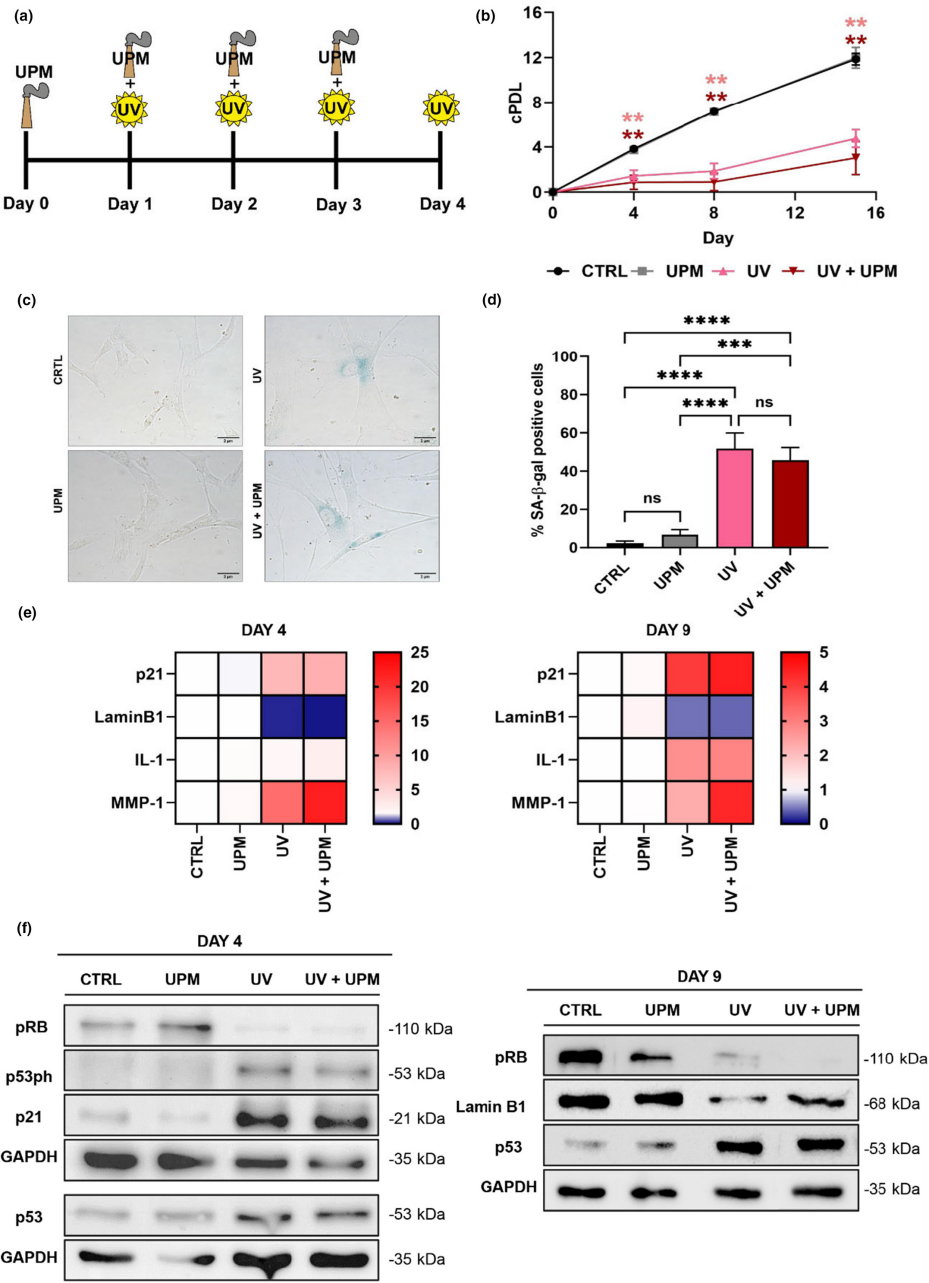
UV + UPM treatment in HFF‐2 causes stress‐induced premature senescence. (a) Schematic representation of the UV + UPM treatment. (b) Growth curve showing cumulative population doublings (cPDL) for each treatment. (c) Representative pictures of SA‐β‐galactosidase assay staining at day 9. (d) Percentage of positive SA‐β‐galactosidase cells. (e) Heatmap representing RT‐qPCR data showing mRNA expression of p21, LaminB1, IL‐1, and MMP‐1 on days 4 and day 9. (f) Composite of western blot pictures showing pRb, p53, p53ph (Serin15), p21, LaminB1, and GAPDH on days 4 and 9. Data represents mean values ± SD, *n* = 3. For statistical analysis one‐way ANOVA was used. In all graphics, ns: non‐significant, *p < 0.05, **p < 0.01, ***p < 0.001, ****p < 0.0001.

Based on our previously established model of UVB‐induced senescence (Greussing et al., 2013), we analyzed the activity of senescence‐associated β‐galactosidase (SA‐β‐gal) on day 9, when senescent cells emerge. We observed that approximately 50% of cells subjected to UV or UV + UPM displayed increased SA‐β‐gal activity in comparison to untreated control (Figure 1c, d). In contrast, no significant changes in SA‐β‐gal activity were observed in UPM‐only‐treated cells. Notably, both UV and UV + UPM‐treated cells on day 9 displayed senescence‐related traits like cell enlargement and flattening (Figure S1B,C), while no morphological changes were observed on UPM‐treated cells. These findings suggest that at the concentration used here (5 μg/mL), UPM treatment alone does not trigger a senescence response or cell death. To further corroborate the observed senescent phenotype observed in UV‐ and UV + UPM‐treated HDF, established senescence markers were investigated. Transcript analysis revealed increased expression of the genes coding for the senescence markers p21, IL‐1, and MMP‐1 along with downregulation of Lamin B1 expression, a common feature in various senescence types (Freund et al., 2012). No changes in the expression of these senescence‐related genes were observed in HDF treated with UPM alone (Figure 1e). Senescence‐associated cell cycle arrest was confirmed by elevated protein levels of both p53 and its phosphorylation at Ser15, a signal of increased protein stability (Cavinato & Jansen‐Dürr, 2017). Moreover, we observed decreased phosphorylation of retinoblastoma protein (pRb) and increased p21 expression following UV and UV + UPM treatments on day 4 (Figure 1f, Figure S1D,E). By day 9, reduced LaminB1 protein expression corroborated the presence of senescent cells in the UV and UV + UPM (Cavinato et al., 2021) treated fibroblasts (Figure 1f, Figure S1C,D). Together, these observations suggest that the selected UV dose effectively induces a bona fide senescence response. Notably, cells treated exclusively with UPM demonstrated no changes in the expression of senescence‐related proteins.

Mitochondrial dysfunction is a key feature of cellular senescence (Gorgoulis et al., 2019; Hutter et al., 2004). Impairment of mitochondrial function can lead to excessive generation of ROS, which in turn can trigger either senescence or apoptosis (Cavinato et al., 2021). Accordingly, we assessed the impact of UPM, UV, and the combination of both stressors on mitochondrial function in HDF. When evaluating mitochondrial network morphology, we observed that the treatment with UPM alone did not result in any morphological alterations in comparison to control untreated cells. However, UV irradiation induced mitochondrial fragmentation, and this effect was significantly intensified by the concomitant treatment with UPM (Figure 2a,b). UV irradiation alone led to notable mitochondrial fragmentation, evidenced by a reduced aspect ratio and a higher end points/branch points ratio, indicating a shift in mitochondrial dynamics (Figure S2A,B). This fragmentation was further amplified when cells were simultaneously treated with UPM, suggesting an intensified impact on mitochondrial structure. Following this structural change, we observed a significant increase in the levels of the fission marker FIS1 under both UV and UV + UPM conditions (Figure 2c,d). In contrast, the fusion marker MFN1 showed no notable variation across these conditions (Figure 2c,e). Furthermore, UV exposure increased mitochondrial ROS levels (Figure 2f), and this effect was significantly enhanced by UV + UPM treatment. UV also led to a reduction in mitochondrial membrane potential (Figure 2g), with a further decrease observed in UV + UPM‐treated cells, although the difference from the UV group did not reach statistical significance.

**FIGURE 2:**
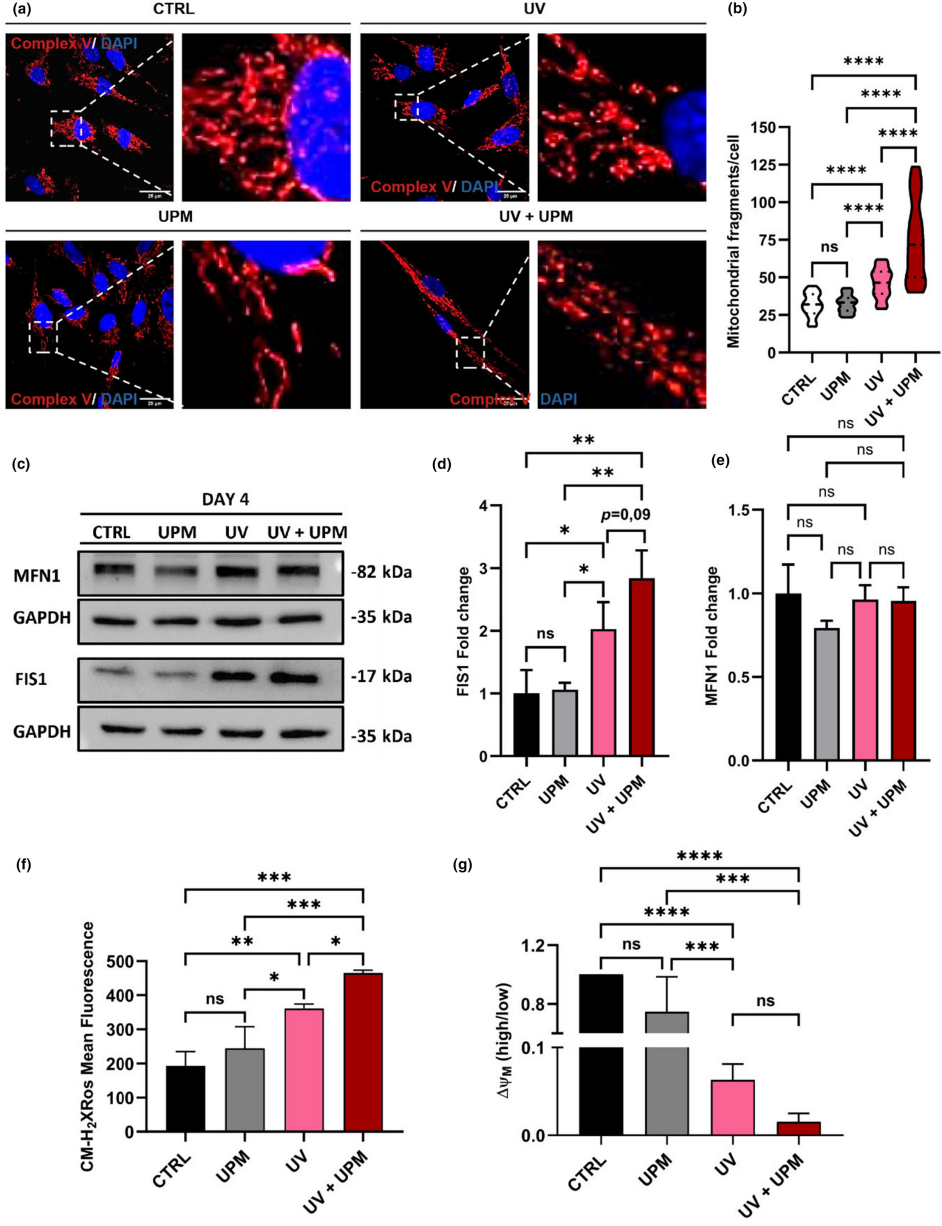
UV + UPM‐treated cells show dysfunctional mitochondria, autophagic flux impairment, and increased apoptosis. (a) Representative pictures from Complex V immunofluorescence on day 4. (b) Measurement of mitochondrial fragments per cell on day 4. (c) Representative western blot pictures showing MFN1, FIS1, and GAPDH on day 4. (d) Densitometry of western blot pictures showing FIS1 on day 4. (e) Densitometry of western blot pictures showing MFN1 on day 4. (f) Fluorescence intensity from CM‐H_2_XRos FACS at day 4. (f) JC‐1 FACS data showing mitochondrial membrane potential on day Data represents mean values ± SD, *n* = 3. For statistical analysis one‐way ANOVA was used. (g) JC1 FACS data showing mitochondrial membrane potential on Day 4. In all graphics, ns: non‐significant, *p < 0.05, **p < 0.01, ***p < 0.001, ****p < 0.0001.

Since increased ROS levels often contribute to DNA damage in human cells, we evaluated the accumulation of DNA damage through immunofluorescence staining using an antibody targeting the phosphorylated form of the histone variant H2X, referred to as γH2AX, known to mark sites of DNA strand breaks. Results revealed that cells treated with UV + UPM had significantly increased DNA damage compared to UV treatment alone (Figure 3a–c), in line with the observed increase of mitochondrial ROS levels.

**FIGURE 3:**
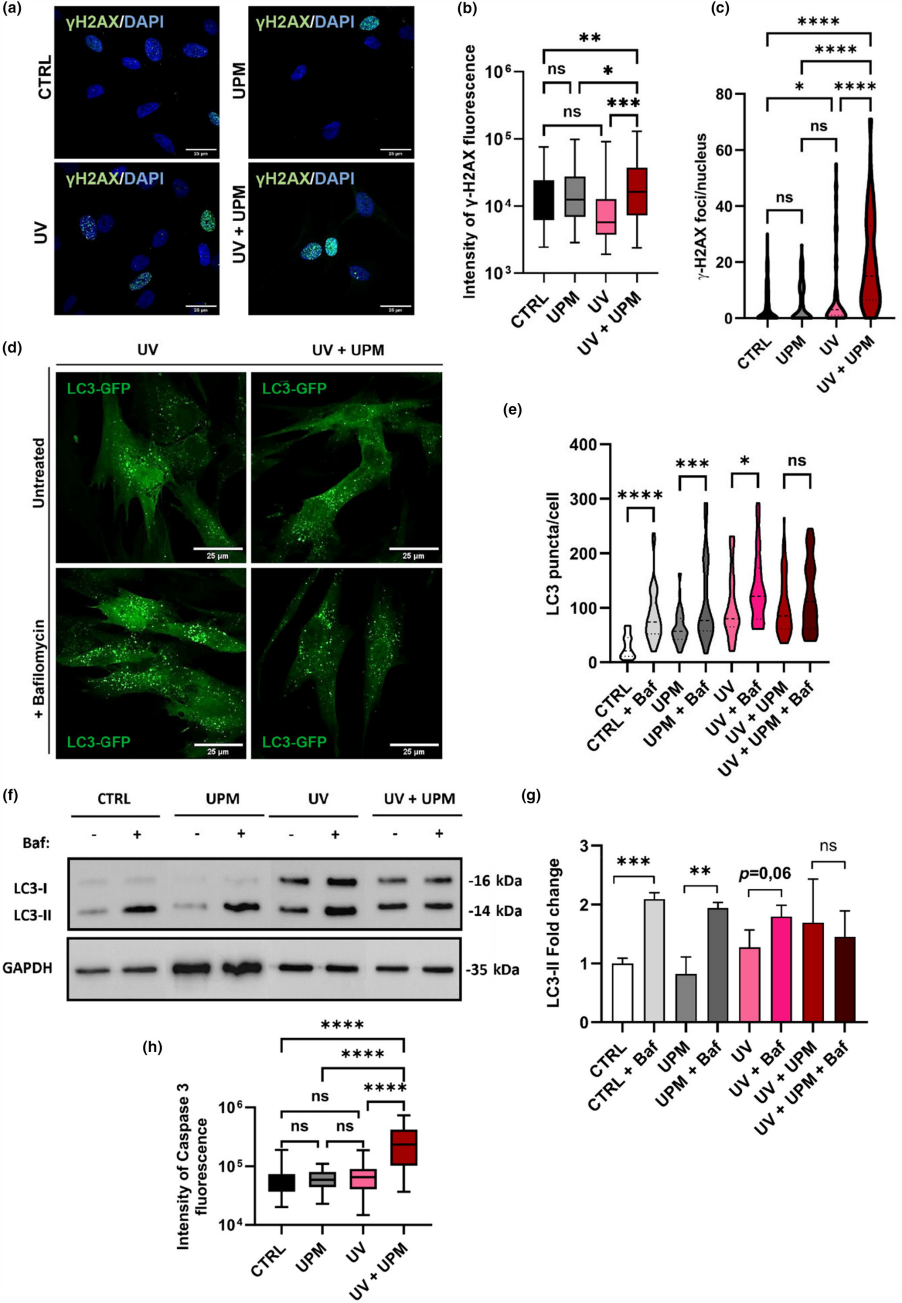
UV + UPM treatment causes DNA damage, autophagic flux impairment and increases apoptosis. (a) Representative pictures from γ‐H2AX immunofluorescence on day 4. (b) Fluorescence intensity from γ‐H2AX immunofluorescence on day 4. (c) Quantification of γ‐H2AX foci per nucleus on day 4. (d) Representative pictures from LC3‐GFP HDF cells subjected to UV, UV + UPM +/− bafilomycin at day 4. (e) Quantification of LC3 puncta/cell from LC3‐GFP HDF subjected to UPM, UV, UV + UPM +/− bafilomycin at day 4. (f) Composite of western blot pictures showing LC3 A/B and GAPDH in HDF posttreatment +/− bafilomycin at day 4. (g) Densitometry of LC3‐II on day 4. (h) Fluorescence intensity from active caspase 3 immunofluorescence at day 4. Data represents mean values ± SD, *N* = 3. For statistical analysis one‐way ANOVA was used. In all graphics ns: non‐significant, *p < 0.05, **p < 0.01, ***p < 0.001, ****p < 0.0001.

Although the role of autophagy in senescence is debated (Kang & Elledge, 2016), autophagy has been shown to play an important role in the survival of senescent HDF generated by UVB irradiation (Cavinato et al., 2016). Thus, we explored the effects of UPM, UV, and the combined UV + UPM treatment on HDF autophagy. Analysis of LC3‐GFP‐positive punctae accumulation revealed a significant increase in the number of autophagosomes following UV and UV + UPM treatment in comparison to UPM‐only treated and untreated control cells. Of note, combined UV + UPM treatment selectively inhibited autophagic flux, as shown by the unchanged number of LC3‐positive puncta following Bafilomycin A treatment (Figure 3d,e, Figure S3A). These observations are further corroborated by our Western blot analysis (Figure 3f,g), reinforcing our conclusions about the altered autophagic response in cells subjected to UV + UPM treatment. The mechanisms underlying the specific impact of UV + UPM on autophagic flux remain to be elucidated and need further investigation.

To assess the impact of impaired autophagic flux on cell fate, we investigated the occurrence of apoptosis induced by UV + UPM treatment using two different approaches. Our findings indicated increased active caspase 3 levels in the UV + UPM condition (Figure 2g, Figure S2D) and a greater percentage of Annexin‐V‐positive cells in UV + UPM compared to cells treated with UV alone (Figure S2E). Previous studies have indicated that under certain conditions, impaired autophagy can lead to an increase in apoptosis, supporting our observations (Ghavami et al., 2014; Lambelet et al., 2018). These results suggest that the decrease in cell numbers in UV + UPM‐treated cells can be attributed to an exacerbated apoptosis on top of the induction of cellular senescence, consistent with our observation that UV + UPM treatment reduced the number of cumulative population doublings (cPDL) without a corresponding increment in the proportion of senescent cells (Figure 1b).

In conclusion, our results suggest that exposure to UV and UPM may accelerate skin aging through multiple mechanisms, including enhancing the presence of senescent fibroblasts and increasing the number of apoptotic cells. This aligns with findings that emphasize the critical role of apoptosis in the aging process (Tower, 2015; Victorelli et al., 2023) recognizing that both senescence and cell death play crucial roles in real‐world aging scenarios and offering a comprehensive perspective by examining these varied cellular responses. We showed that the concomitant treatment of UV and UPM increases DNA damage, mitochondrial dysfunction, oxidative stress, and led to impaired autophagy. This synergistic interaction between UV and UPM exacerbates their detrimental effects, as both are known to stimulate the production of ROS species. Additionally, the combination of these stressors disrupts autophagy flux, further compromising cell viability. While potential damage of UPM in conjunction with UV to other organelles apart from mitochondria remains elusive, the disturbance in autophagy flux could potentially be attributed to lysosomal damage (Guerrero‐Navarro et al., 2022). As such, future research will be required to determine if lysosomal membrane integrity is compromised by UV + UPM combined treatment, which may trigger the discovery of new potential targets to limit skin damage upon exposure to UVB and UPM.

## AUTHOR CONTRIBUTIONS

L.G.‐N., P.J.‐D., and M.C. designed the study and the experiments to be performed. L.G.‐N. performed the experiments and analyzed the data. M.C. supervised the work. L.G.‐N. and M.C. wrote and prepared the draft. P.J.‐D. and M.C. provided critical commentary and corrected the text. P.J.‐D. acquired funding. All authors have read and agreed to the published version of the manuscript.

## FUNDING INFORMATION

The research was performed under the ARDRE COFUND doctoral training program, funded by the European Commission Horizon 2020 Marie Sklodowska‐Curie research grant number 847681. Work in the Jansen‐Dürr/Cavinato lab was supported by the Austrian Science Funds (FWF), with funds from the “Fonds‐Zukunft Österreich” in the frame of the Research Group “Targeting cellular senescence based on inter‐organelle communication, multilevel proteostasis, and metabolic control” (SENIOPROM, project #FG2400B) and by the Austrian Science Funds (FWF), P315820.

## CONFLICT OF INTEREST STATEMENT

The authors declare no conflict of interest.

## Supporting information


Data S1.

## Data Availability

The data can be obtained from the corresponding author upon a reasonable request.
