# Supplementary material for: Synergistic interplay of UV radiation and urban particulate matter induces impairment of autophagy and alters cellular fate in senescence‐prone human dermal fibroblasts
Source: Aging Cell. 2024 Jan 12;23(4):e14086. doi: 10.1111/acel.14086 (PMC11019139; doi:10.1111/acel.14086)
Supplement: Supplementary file 1 — Data S1. [file ACEL-23-e14086-s001.docx]

**Supplementary material**

**Materials and Methods**

**Chemicals:**

All chemicals were purchased from Sigma (Steinheim, Germany) unless stated otherwise.

**Cell culture and UV+UPM treatment**

Human diploid fibroblasts derived from newborn foreskin ( HFF-2, #SCRC-1042, ATCC® Manassas, VA,) were cultured in Dulbecco’s Modified Eagle Medium (DMEM) supplemented with 10% foetal bovine serum (FBS), 4mM L-glutamine and 1% penicillin-streptomycin. Cumulative population doublings were calculated as previously described (Greussing et al., 2013).

HFF-2 cells were initially placed onto 10cm plates at varying densities: 6x10^5^ cells/plate for irradiated cells and 3x10^5^ cells/plate for non-irradiated ones. For UV irradiation, cells were rinsed with Hank's balanced salt solution (HBSS) and then covered with 2.5 mL of the same solution. To administer UVA (7J/cm^2^) and UVB (0.05J/cm^2^) irradiation, the Bio-Sun System was used, once a day from day 1 to day 4, for both UV+UPM and UV treatments. In the case of UPM and UV+UPM treated cells, UPM treatment was initiated on day 0 and concluded on day 3. For UV+UPM, UPM (5µg/mL) was added to the medium on day 0 and post irradiation from day 1 to day 3.

**Cell surface area measurement**

Cell surface area was measured from at least 50 cells per group using ImageJ Software as described (Wedel et al., 2020).

**Cytochemistry for Senescence-Associated-β-galactosidase (SA-β-Gal)**

SA-β-galactosidase staining was performed as described (Greussing et al., 2013). At least 100 cells were examined in each sample, and the ratio of SA-β-galactosidase positive cells was determined by dividing the count of blue cells by the total cell count.

**Immunoblotting**

Protein lysates were prepared with modified RIPA buffer as previously described (Cavinato et al., 2016). The concentration of protein was determined using the Pierce® BCA Protein Assay Kit following the manufacturer's protocol. Protein lysates were separated on SDS-PAGE and transferred to PVDF membranes as described (Wedel et al., 2020). The membranes were blocked in 5% non-fat dry milk in PBS-Tween and incubated with the following primary antibodies: Phospho-Rb Ser807/811 (#9308, Cell signaling), Lamin B1 (ab16048, Abcam), p53 (DO-1): sc-126 (Santa Cruz), Phospho-p53 Ser15 (#9284, Cell Signaling), p21 Waf1/Cip1 (12D1) (#2947, Cell Signaling), LC3 A/B (#4108S, Cell Signaling), FIS1 (#10956-1-AP, Proteintech), MFN1 (#AB57602, Abcam), GAPDH (0411): sc-47724, (Santa Cruz). The blots were then incubated with a secondary antibody conjugated with horseradish peroxidase (HRP), incubated in luminol developer solution (Immobilon Western, HRP Substrate, Millipore, Burlington, MA, USA) and exposed in the ChemiDoc^TM^ Imaging System (Bio-Rad). The protein levels were quantified using ImageJ Software, and the intensity of the bands was normalized to the loading control protein GAPDH.

**RNA isolation, cDNA synthesis and qPCR quantification**

RNA was extracted using the RNeasy® Mini Kit by Qiagen, following the manufacturer's protocol. The concentration of RNA was measured using the Nanodrop 2000 system (Thermo Scientific). To obtain cDNA, the RNA was reverse transcribed using the High Capacity cDNA Reverse Transcription Kit (Applied Biosystems by Thermo Fisher Scientific, Vienna, Austria) according to the manufacturer's instructions. For qPCR analysis, each sample was run in triplicates using AceQ® Universal SYBR® Green qPCR Master Mix (Vazyme, Q511-02) on a QuantStudio™ 7 Flex Real-Time PCR System (Thermo, 4485701). The following primer pairs were employed: p21 FW primer: GGG ACA GCA GAG GAA GAC C; p21 RE primer: GGC GTT TGG AGT GGT AGA AA; LaminB1 FW primer: AAG CAG CTG GAG TGG TTG TT; LaminB1 RE primer: TTG GAT GCT CTT GGG GTT C; IL1α FW primer: TCA GCA AAG AAG TCA AGA TGG C; IL1α RE primer: CAT GGA GTG GGC CAT AGC TT; MMP1 FW primer: CAT CGT GTT GCA GCT CAT GA; MMP1 RE primer: ATG GGC TGG ACA GGA TTT TG; B2M FW primer: GAA TTC ACC CCC ACT GAA AA; B2M RE primer: CTC CAT GAT GCT GCT TAC A. The housekeeping gene B2M was used for normalization.

**Determination of cell cycle stages proportions**

For assessing cell proliferation, we employed the 5-bromo-2-deoxy-uridine (BrdU) labeling and detection kit I (ref 11296736001, Roche), following the manufacturer's protocol. Post-labeling, cells were co-stained with DAPI to visualize the nuclei. The fluorescence intensity of both DAPI and BrdU was quantified. Based on the fluorescence intensity, nuclei were categorized into distinct phases of the cell cycle: cells with low DAPI and low BrdU fluorescence intensity were classified as being in the G1 phase; those exhibiting high BrdU fluorescence were identified as being in the S phase; and cells with high DAPI but low BrdU fluorescence intensity were categorized as being in the G2 phase.

**Determination of cell death with AnnexinV/PI**

AnnexinV/PI staining was used to detect apoptotic and necrotic cells, following the manufacturer’s protocol (FITC Annexin V Apoptosis Detection Kit I, BD Pharmingen™, Vienna, Austria). The fluorescence signals were measured with a BD FACS Canto II flow cytometer, with measurements performed in triplicate for each group. The percentage of apoptotic cells was calculated as the sum of necrotic (Annexin V-negative/PI-positive), early (Annexin V-positive/PI-negative), and late-stage (Annexin V-positive/PI-positive) apoptotic cells.

**Determination of mitochondrial membrane potential with JC-1**

The fluorescent probe JC-1 was utilized to determine the mitochondrial membrane potential. HFF-2 cells were trypsinized, stained with 0.5 μg/ml JC-1 solution, and incubated at 37°C for 30 minutes. Following incubation, the cells were washed, and the fluorescence was analyzed using a BD FACS Canto II flow cytometer. To serve as a positive control, cells were treated with 5 μM Carbonyl cyanide-4-(trifluoromethoxy) phenylhydrazone (FCCP). For the evaluation of the results a ratio was obtained by dividing the percentage of mitochondria with high membrane potential by the percentage of mitochondria with low membrane potential of a given population.

**Determination of mitochondrial fragmentation**

To analyze mitochondrial fragmentation, we used Complex V immunofluorescence images. The aspect ratio (major to minor axis) and end points/branch points of the mitochondrial network were quantitatively assessed from these images. The aspect ratio was calculated to evaluate mitochondrial elongation or fragmentation, while the ratio of endpoints (terminal points of a tubule) to branch points (junctions where a tubule bifurcates) provided insight into the network's complexity. The analysis was conducted on ImageJ following guidelines in reference (De Vos & Sheetz, 2007).

**Determination of mitochondrial ROS with CM-H2XRos**

To determine mitochondrial ROS levels, the cells were stained with 100 nM CM-H2XRos (Molecular Probes, Vienna, Austria) for 30 minutes at 37 °C after trypsinization. Following staining, the cells were washed with PBS, and fluorescence was measured using a BD FACS Canto II flow cytometer. A positive control was established by treating the cells with 0.5 μM rotenone.

**Immunofluorescence**

Cells were seeded on glass coverslips, fixed with 4% paraformaldehyde, permeabilized in PBS containing 0.1% sodium citrate and 0.3% Triton-X, and blocked with 1% bovine serum albumin in PBS. The cells were then incubated with primary antibodies against γH2AX (#2577S, Cell Signaling), active Caspase-3 (#559565, BD), Complex V – subunit β (#A21351, Invitrogen) overnight at 4°C. Alexa Fluor anti-rabbit 488 or Alexa Fluor anti-mouse 546 were used as secondary antibodies. The nuclei were counterstained with 4', 6-diamidino-2-phenylindole (DAPI) and the cells were analyzed using Cell Voyager CV1000 Yokogawa (Visitron Systems).

Quantification of nuclear γH2AX fluorescence intensity and number of foci, active caspase-3 fluorescence intensity and Complex V positive particles per cell was performed using ImageJ Software.

**LC3-GFP stable overexpression**

Expression vectors carrying the human LC3B gene fused to the 5' end of the GFP gene and lentiviral particles carrying the expression vectors were generated following the procedure described in reference (Cavinato et al., 2016). LC3-GFP-expressing HFF-2 cells were generated via lentiviral transduction.

**Statistics**

All experiments were performed with a minimum of three independent biological replicates. The results are presented as the means ± standard deviation of these experiments. To determine significant differences between experimental conditions, the one-way ANOVA test was used. The significance level for all graphs is indicated as follows: ns: non-significant, *p < 0.05, **p < 0.01, ***p < 0.001, ****p < 0.0001.

**Supplementary Material**


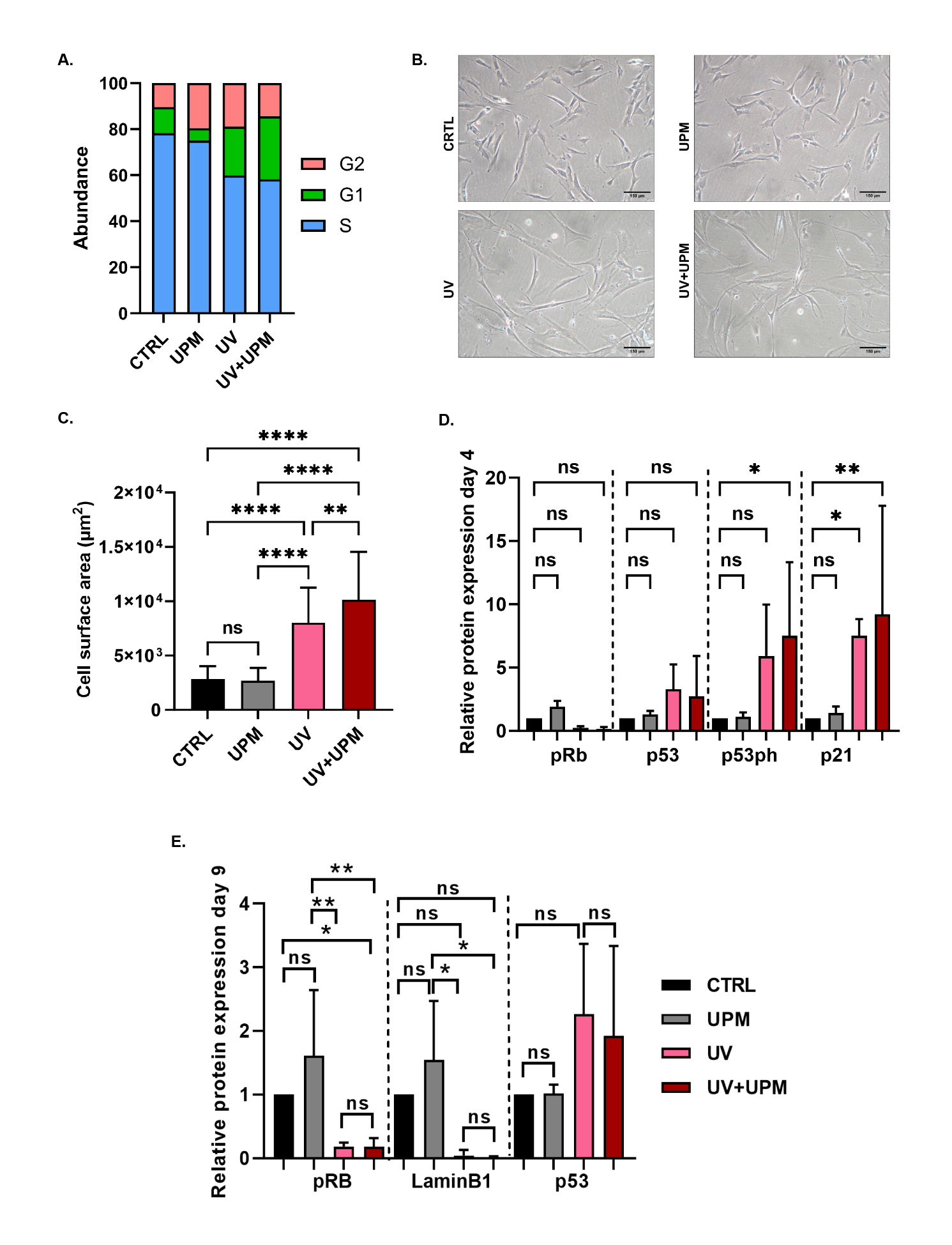


**Supplementary Figure 1. Enhanced Cell Area and Upregulated Senescence-Associated Protein Expression Induced by UV+UPM Treatment** **A**. Percentage of cells in different phases of the cell cycle at day 4. **B**.Representative pictures from cells on day 9. **C.** Measurement of cell surface area on day 9. **D.** Densitometry of western blot pictures showing pRb, p53, p53ph (Serin15) and p21 on day 4. **E.** Densitometry of western blot pictures showing pRb, LaminB1 and p53 on day 9. Data represents mean values ± SD, N=3. For statistical analysis one-way ANOVA was used. In all graphics ns: non-significant, *p < 0.05, **p < 0.01, ***p < 0.001, ****p < 0.0001.


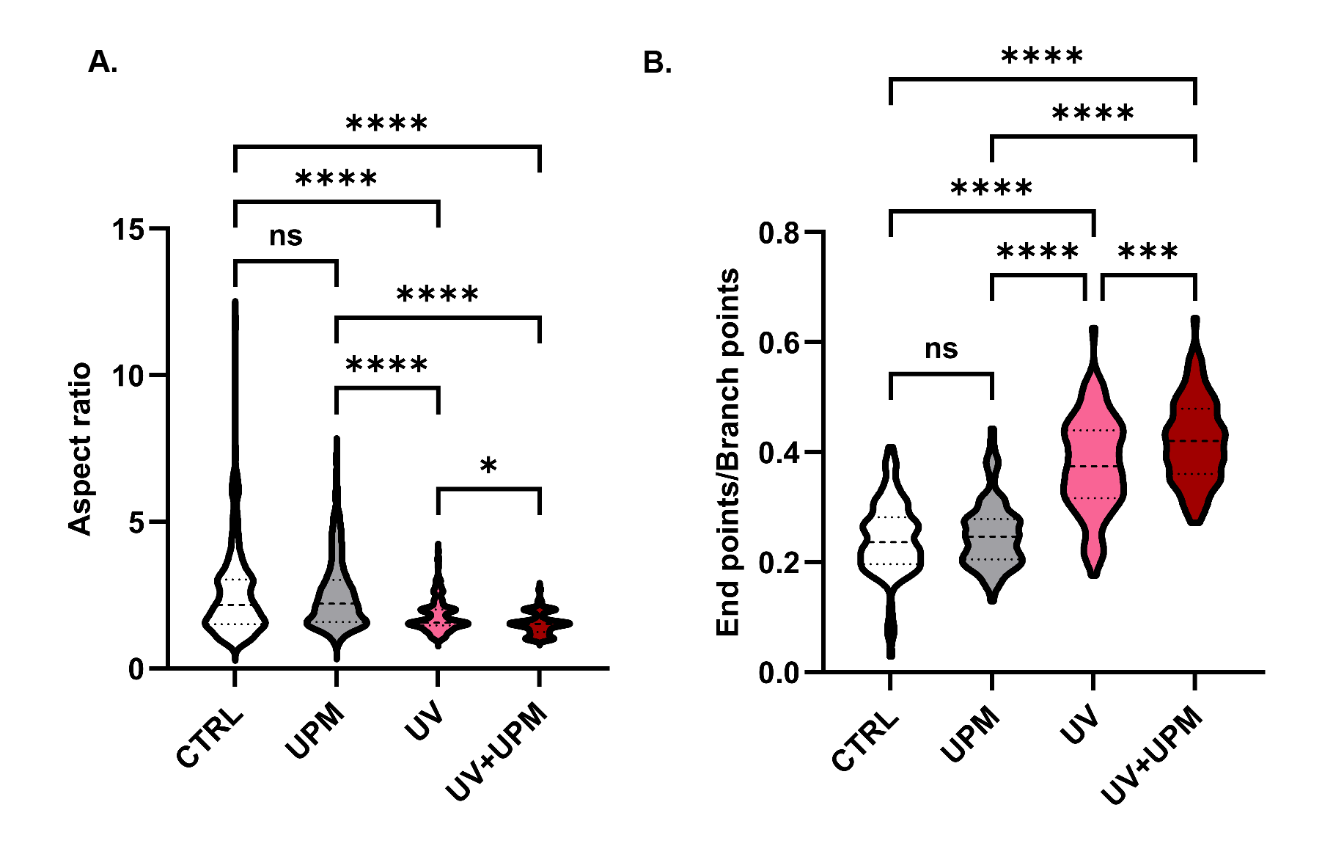


**Supplementary Figure 2: Mitochondrial dysfunction Induced by UV+UPM Treatment. A.** Quantification of mitochondria aspect ratio (Comple V IF presented in Fig. 2A) at day 4. **B** Quantification of End points/branch points from Complex V images at day 4. Data represents mean values ± SD, N=3. For statistical analysis one- way ANOVA was used. In all graphics ns: non-significant, *p < 0.05, **p < 0.01, ***p < 0.001, ****p < 0.0001.


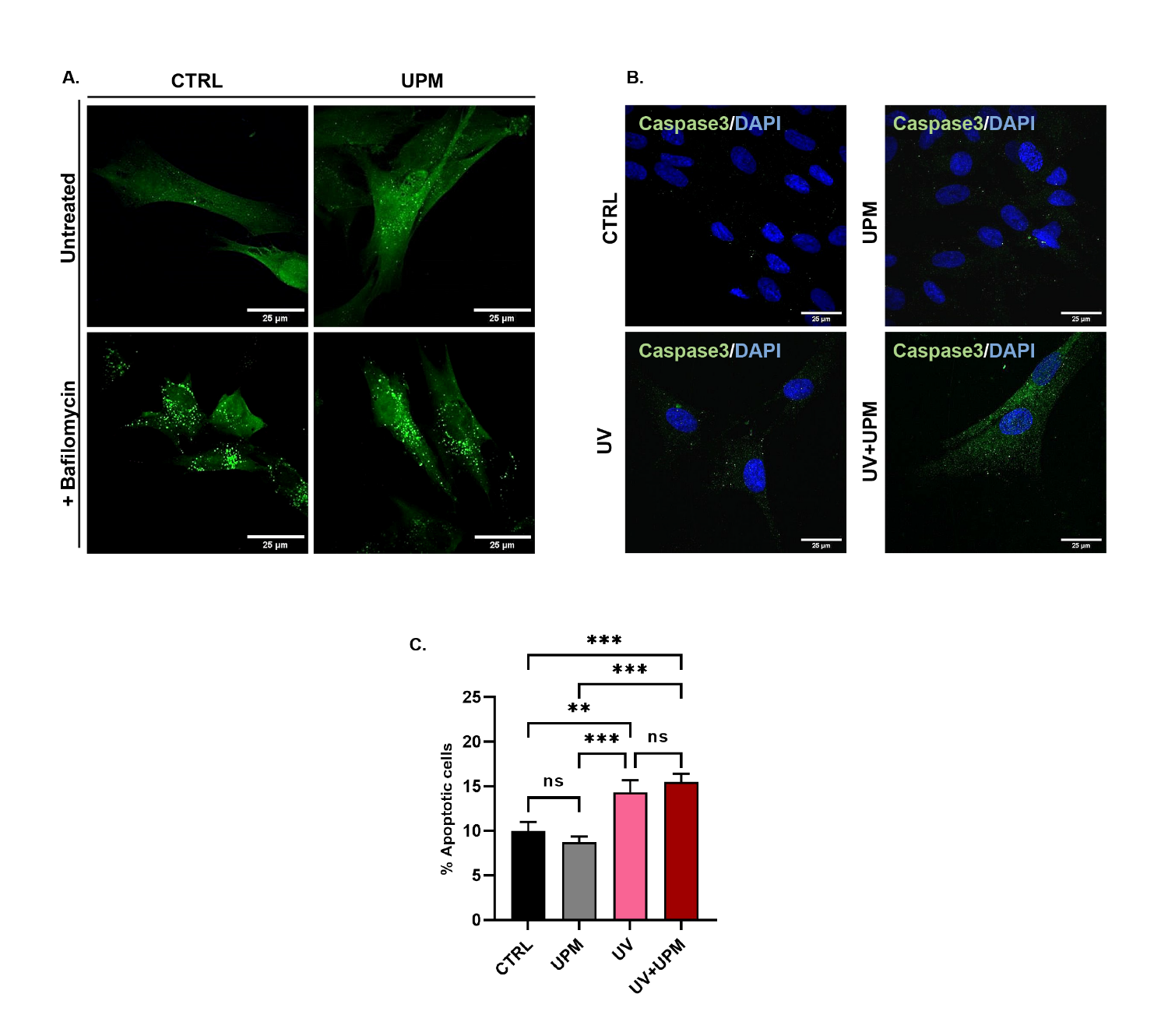


**Supplementary Figure 3: Autophagic flux in untreated and UPM treated cells and enhanced apoptosis produced by UV+UPM treatment. A.** Representative pictures from LC3-GFP HDF cells untreated or subjected to UPM +/- Bafilomycin on day 4. **B.** Representative pictures from active caspase 3 immunofluorescence on day 4. **C.** Percentage of apoptotic cells from AnnexinV-PI FACS at day 4. Data represents mean values ± SD, N=3. For statistical analysis one- way ANOVA was used. In all graphics ns: non-significant, *p < 0.05, **p < 0.01, ***p < 0.001, ****p < 0.0001.
